# Peak calling by Sparse Enrichment Analysis for CUT&RUN chromatin profiling

**DOI:** 10.1186/s13072-019-0287-4

**Published:** 2019-07-12

**Authors:** Michael P. Meers, Dan Tenenbaum, Steven Henikoff

**Affiliations:** 1 Basic Sciences Division, Fred Hutchinson Cancer Research Center, 1100 Fairview Ave N, Seattle, WA 98109 USA; 2 Scientific Computing, Fred Hutchinson Cancer Research Center, 1100 Fairview Ave N, Seattle, WA 98109 USA; 3 Howard Hughes Medical Institute Research Laboratory, Seattle, USA

**Keywords:** CUT&RUN, Epigenome profiling, Peak calling

## Abstract

**Background:**

CUT&RUN is an efficient epigenome profiling method that identifies sites of DNA binding protein enrichment genome-wide with high signal to noise and low sequencing requirements. Currently, the analysis of CUT&RUN data is complicated by its exceptionally low background, which renders programs designed for analysis of ChIP-seq data vulnerable to oversensitivity in identifying sites of protein binding.

**Results:**

Here we introduce Sparse Enrichment Analysis for CUT&RUN (SEACR), an analysis strategy that uses the global distribution of background signal to calibrate a simple threshold for peak calling. SEACR discriminates between true and false-positive peaks with near-perfect specificity from “gold standard” CUT&RUN datasets and efficiently identifies enriched regions for several different protein targets. We also introduce a web server (http://seacr.fredhutch.org) for plug-and-play analysis with SEACR that facilitates maximum accessibility across users of all skill levels.

**Conclusions:**

SEACR is a highly selective peak caller that definitively validates the accuracy of CUT&RUN for datasets with known true negatives. Its ease of use and performance in comparison with existing peak calling strategies make it an ideal choice for analyzing CUT&RUN data.

**Electronic supplementary material:**

The online version of this article (10.1186/s13072-019-0287-4) contains supplementary material, which is available to authorized users.

## Background

Eukaryotic DNA is wrapped in millions of nucleosomes that restrict access of thousands of DNA binding proteins, including transcription factors (TFs) that bind to enhancers and promoters to activate or repress gene expression and often specify cell fate [1]. Determining where chromatin and DNA binding proteins localize in the genome is crucial for elucidating fundamental principles of genome regulation. Efforts to map DNA binding proteins to their targets in the genome originated with chromatin immunoprecipitation (ChIP), in which proteins are physically crosslinked to their targets, immunoprecipitated with protein-specific antibodies, and their crosslinks reversed for downstream analysis of associated DNA [2]. In recent years, such techniques have been adapted for high-throughput sequencing readouts [3–5], which has enabled genome-wide identification of thousands of binding sites for hundreds of proteins [6]. This proliferation of data is accompanied by a need for fast and accurate analysis tools to process them.

Standard ChIP-seq data analysis often involves “peak calling” algorithms, which identify genomic regions at which target ChIP-seq signal is enriched in comparison with background noise from a control DNA input or non-targeting antibody experiment [7]. Such algorithms frequently employ Poisson or negative binomial models of global and local read distributions to derive statistical measures of signal enrichment over background, and extensive efforts have been dedicated to evaluating the merits of such design choices [8]. Importantly, since ChIP-seq experiments are typically sequenced deeply and thus feature high background, most peak calling algorithms designed for the analysis of ChIP-seq data use models that are optimized primarily for high recall to distinguish signal from noise [9].

In contrast with ChIP-seq, CUT&RUN is an in situ epigenome profiling technique that uses an antibody-targeted micrococcal nuclease (MNase) fusion protein to selectively digest and liberate DNA fragments at sites of protein binding while leaving the remainder of the genome behind; thus, it features exceedingly low background in comparison with ChIP-seq [10, 11]. The low read depths and background levels of CUT&RUN data render standard peak callers vulnerable to reduced precision (i.e., avoidance of false positives) due to the sparseness of the background, resulting in any spurious background read being called as a peak. Thus, rather than requiring highly sensitive methods to distinguish signal from background noise, peak calling from CUT&RUN data requires high specificity for true positive peaks.

Here we introduce Sparse Enrichment Analysis for CUT&RUN (SEACR), a peak caller designed for the processing of paired-end CUT&RUN data. SEACR is model free and empirically data driven and therefore does not require arbitrary selection of parameters from a statistical model. Moreover, we show that SEACR retains superior selectivity versus common ChIP-seq peak callers from CUT&RUN data, while retaining competitive performance across a range of experimental configurations. Finally, we have made SEACR available to the community through a simple web interface. We conclude that SEACR is fast, accurate, scalable and simple to use for the analysis of CUT&RUN data.

## Results

### Peak calling based on fragment block aggregation

**Fig. 1:**
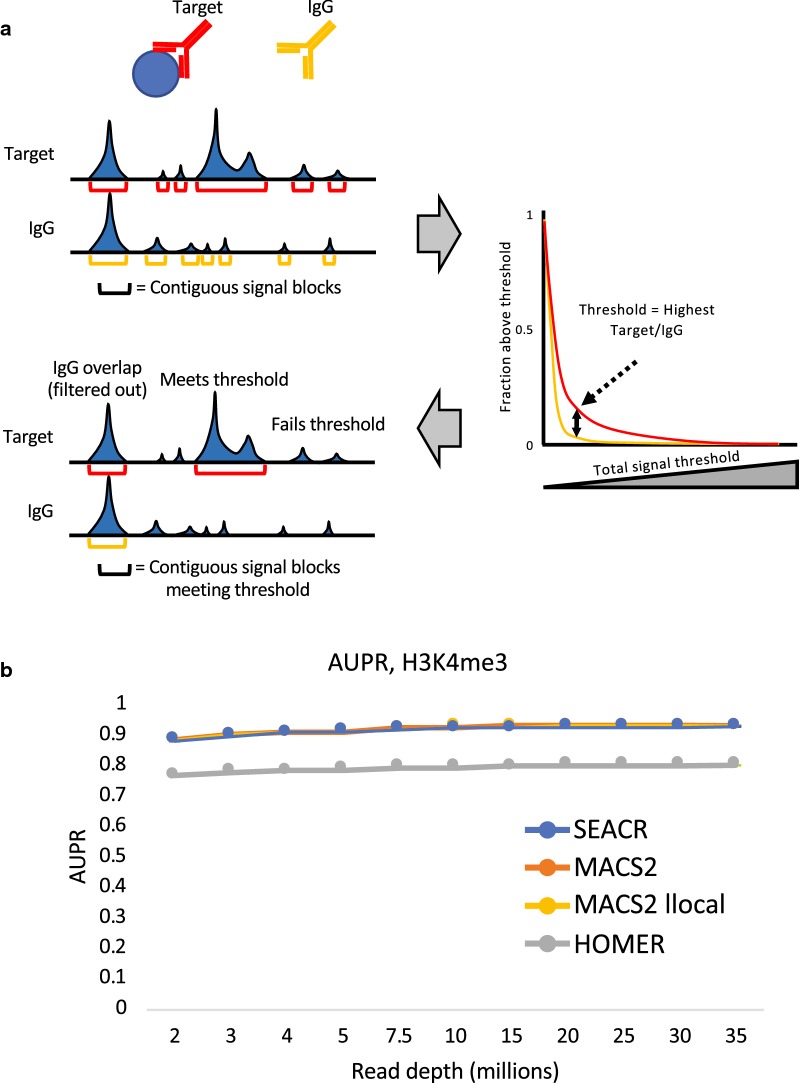
SEACR enforces peak calling specificity across a range of read depths. **a** Schematic of SEACR methodology. Contiguous signal blocks (top) are identified and plotted by the percentage of blocks exceeding a total signal threshold (right), and an optimal threshold is empirically identified and used for filtering (bottom). **b** Plot of area under the precision–recall curve (AUPR) for peaks called from H3K4me3 CUT&RUN data by SEACR, MACS2, MACS2 with local lambda inactivated (MACS2 llocal), and HOMER that were compared to a stringent test set of H3K4me3 peaks called from ENCODE ChIP-seq data. Read subsampling levels are indicated on the X-axis

To address the problem of oversensitivity in CUT&RUN peak calling, we developed Sparse Enrichment Analysis for CUT&RUN (SEACR), a peak calling algorithm that enforces precision from sparse data by quantifying the global distribution of background signal and using it to set a stringent empirical threshold for peak identity. CUT&RUN data from target antibody and IgG control experiments are first parsed into signal blocks representing segments of continuous, nonzero read depth by fragment spanning read pairs, and the signal in each block is calculated by summing read counts (Fig. 1a). A plot of the proportion of signal blocks in target versus IgG (y-axis) is used to identify the threshold value at which the percentage of target versus IgG blocks is maximized; then target blocks failing to meet the threshold are filtered out, leaving enriched peaks (Fig. 1a). We also filtered out blocks that overlap an IgG block meeting the threshold as a means to eliminate spurious peaks that arise either through multiple mapping at repeated regions or at false-positive sites [12].

We evaluated the effectiveness of using signal blocks as a metric for discrimination between potential true- and false-positive peaks in comparison with common strategies employed by ChIP-seq peak callers. For CUT&RUN datasets profiling K562 cells for H3K4me2, H3K4me3, H3K27me3, and CTCF at several different read subsampling levels, we used SEACR’s block aggregation utility to sort all signal blocks in the genome by total signal, and in parallel called peaks using MACS2 [13] or HOMER [14] with maximally relaxed peak cutoffs as detailed in the "Methods" section. We then used comparisons with validated ChIP-seq peak calls from ENCODE to plot precision–recall curves for each subsampled dataset for each of the three cutoff strategies (total signal in signal block for SEACR, -log_10_(FDR) for MACS2, and “Peak Score” for HOMER) and calculated areas under the curve (AUPR). SEACR AUPR was competitive with MACS2 and HOMER for all datasets interrogated and outperformed both of them across all read subsampling levels for CTCF (Fig. 1b, Additional file 1: Fig. S1A–C). Notably, though running MACS2 without a local lambda parameter to imitate the non-local peak identification of SEACR improved performance of MACS2 peak calling for H3K27me3 data, it made a negligible difference in performance for H3K4me2, H3K4me3 and CTCF. These results confirm that total signal in signal blocks is a valid metric for discriminating enriched regions from CUT&RUN data.

### SEACR thresholding validates “gold standard” CUT&RUN data

**Fig. 2:**
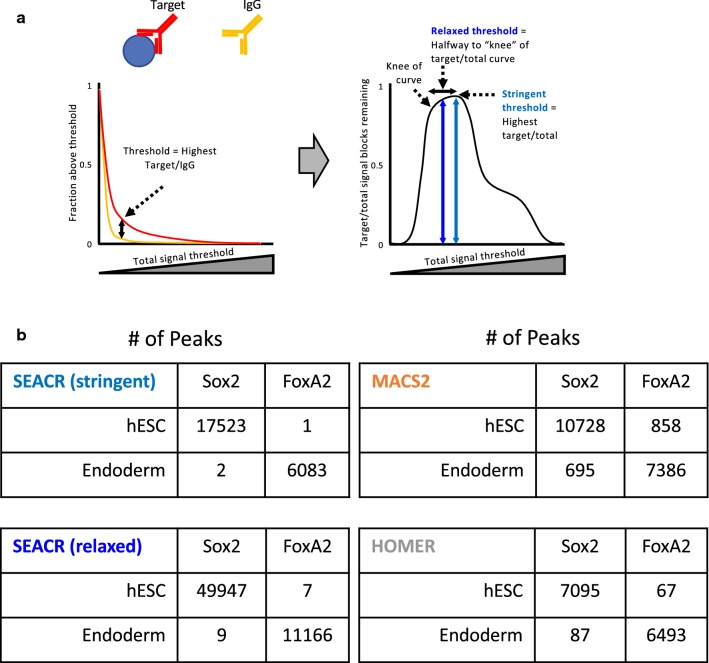
SEACR minimizes true negative detection from a gold standard dataset. **a** Schematic of SEACR threshold selection. A target/total signal block curve (right) is calculated, and the stringent and relaxed thresholds are set based on the knee and the peak of the curve. **b** Peaks were called from Sox2 or FoxA2 experiments carried out in either hESCs or Endoderm cells using SEACR in stringent or relaxed mode; MACS2; or HOMER

Peak calling programs often feature several options for parameter selection, which afford flexibility to adapt to specific use cases, but also complicates analysis for users. Thus, we introduced only two major options for SEACR. First, we offer the option of either providing a control IgG dataset or choosing a global threshold value (default = IgG). The provision of a control IgG dataset is recommended for general use and is used in the remainder of the manuscript. Second, we implemented “stringent” and “relaxed” peak selection modes (default = stringent), which correspond to the threshold at which the maximum percentage of target versus IgG signal blocks are retained, or a threshold halfway between the maximum and the “knee” of the target percentage curve, respectively (Fig. 2a).

To test how well SEACR can avoid false-positive peaks in a CUT&RUN dataset in which such distinctions are known, we used SEACR, MACS2, and HOMER to call peaks from CUT&RUN data for two transcription factors (TFs), Sox2 and FoxA2 in human embryonic stem cells (hESCs) and definitive endoderm (DE) cells. Sox2 expression is restricted to hESCs, and FoxA2 expression is restricted to DE cells [15]. All three methods called a comparable number of peaks for Sox2 in hESCs or FoxA2 in DE cells. In contrast, SEACR called only 1–2 peaks for each factor when it is not expressed (Fig. 2a, “stringent”). HOMER and MACS2 called up to ~ 900 spurious peaks in these datasets using default peak calling thresholds for each program; these trends held when analyzing total bases covered by peaks or percentage of reads in peaks (Fig. 2—Additional file 2: Fig. S2A, B, “stringent”). Notably, this high selectivity of SEACR was maintained when running in “relaxed” mode (Fig. 2b, Additional file 2: Fig. S2A, B, “relaxed”). These results indicate that SEACR outperforms popular ChIP-seq peak callers in avoiding false-positive peak calls. Indeed, the combination of CUT&RUN data with SEACR peak calling results in nearly complete exclusion of false positives, which validates the trustworthiness of the CUT&RUN method.

### SEACR default thresholds are robust over a wide range of read depths

**Fig. 3:**
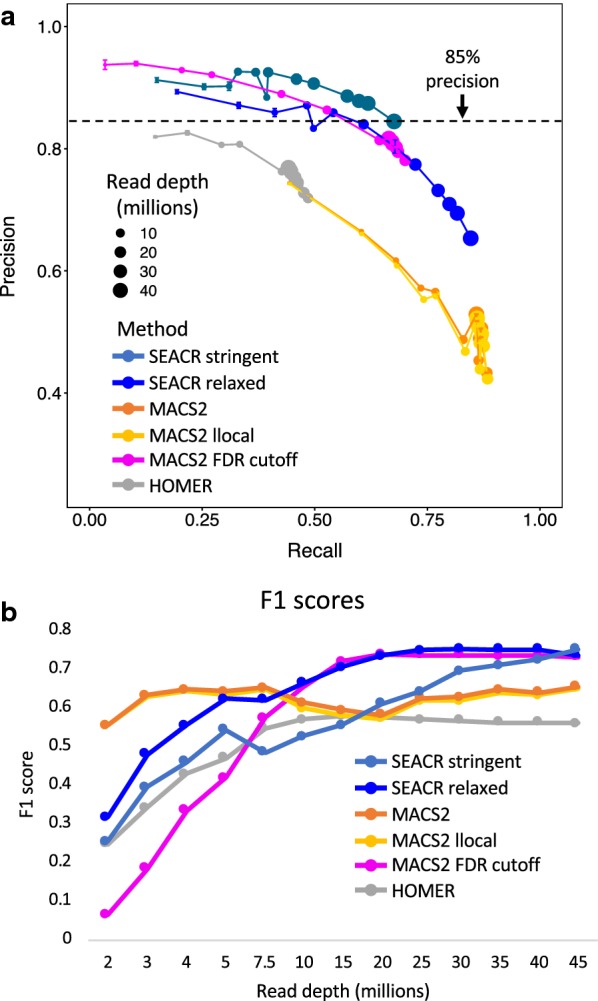
SEACR thresholding provides an improved specificity/sensitivity trade-off. **a** Precision–recall curves derived from a comparison of peaks called from subsampled H3K4me2 CUT&RUN to ENCODE peaks called from H3K4me2 ChIP-seq data. Colors denote peak calling method, size of points denotes read subsampling level, and dotted black line denotes 85% precision in peak calling. **b** F1 score for each point on the plots in A

To test the performance of SEACR default thresholds in comparison with thresholds set by ChIP-seq peak callers at low read depths, we called peaks from H3K4me2 CUT&RUN data subsampled 10 times each at 12 different read depths spanning from 2 million to 45 million reads. We used default thresholds for SEACR with both stringent and relaxed modes, MACS2 in standard or local lambda-inactivated “narrow peak” mode, and HOMER in “factor” mode. We then compared peaks with peaks called by the ENCODE consortium using MACS2 on ENCODE ChIP-seq data, assigning ENCODE peaks meeting a −log_10_ FDR threshold of greater than 10 as a stringent “truth set”. In both stringent and relaxed modes, SEACR consistently maximized the fraction of called peaks that were inside the test set (precision), with all tests in stringent mode and most in relaxed mode surpassing 85% precision (Fig. 3a). In contrast, all tests using MACS2 or HOMER with default cutoffs failed to reach the same precision. This indicates that the precision of SEACR is robust across a range of read depths.

To analyze the general optimality of combined recall and precision for each peak caller, we calculated the F1 score for each peak caller at each read subsampling level, such that larger F1 scores corresponded with higher performance in a combination of the two metrics. SEACR relaxed mode exhibited superior performance at all subsampling levels above ~ 7.5 million reads (Fig. 3b, blue curve). To account for the fact that peak callers such as MACS2 have parameters that can be optimized to adjust the desired precision–recall balance, we selected a stringent set of peaks from the MACS2 peak calls that meet a −log_10_(FDR) threshold of greater than 10, and recalculated F1 scores in comparison with SEACR. Although the more stringent MACS2 peak calls had improved performance above 10 million fragments, performance suffered at fragment subsampling levels below 10 million reads, rendering SEACR superior at those levels (Fig. 3b, magenta curve). Therefore, SEACR thresholds remain competitive with widely used ChIP-seq peak callers across multiple parameter selection strategies, even in the absence of arbitrary user input for the purposes of optimization. Although our conclusions are based on the presumption that high-scoring ENCODE peaks are true positives, the fact that they were originally called using MACS2 leads us to expect that the superior performance of SEACR on CUT&RUN data will generalize to any set of true positives. Thus, SEACR is an accurate peak caller for CUT&RUN data across a range of read depths and maintains a high percentage of true positive peak calls at low read depth.

### SEACR retains broad domain structures

**Fig. 4:**
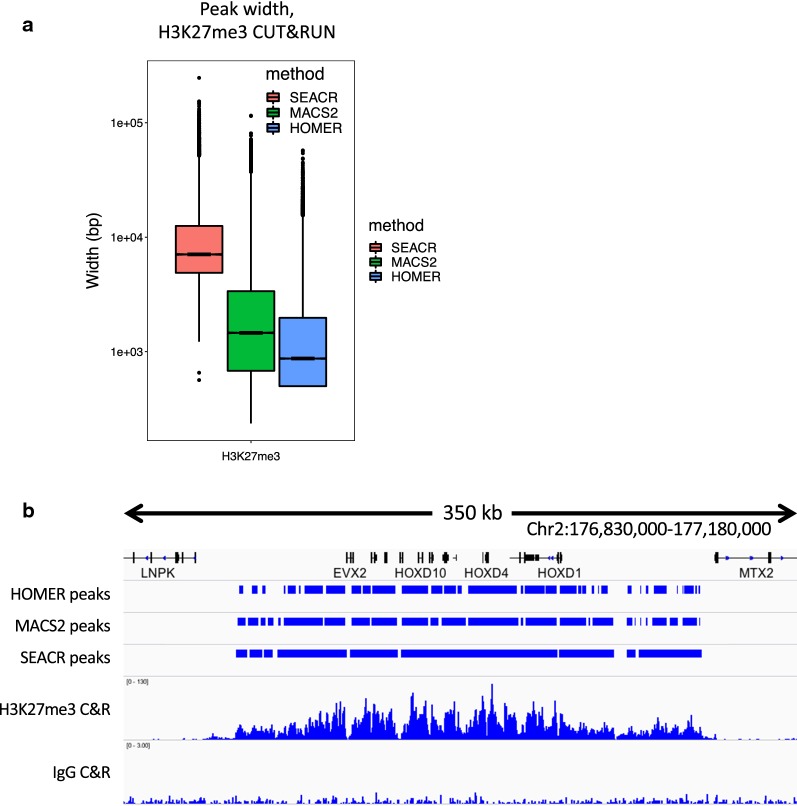
SEACR identifies coherent broad domains. **a** CUT&RUN domains identified by SEACR are broader than those found by MACS2 or HOMER. **b** A representative region of a H3K27me3 profile is shown. SEACR domains are relatively coherent compared with HOMER and MACS2 domains, which are fragmented into mixtures of wide and narrow segments

Peak calling is often confounded by the diverse distributions of chromatin proteins and modifications; for instance, transcription factors are expected to cluster at narrow genomic loci in peaks, whereas many histone modifications such as H3K27me3 cover broad regions that are not easily summarized by methods that detect peaks. Since our signal block approach is agnostic to region width, we reasoned that SEACR might be equally successful at identifying broad domains as it is for the peaks identified from Sox2, FoxA2, and H3K4me2 data. To test performance on broad domains, we called peaks using SEACR, MACS2, and HOMER (using “stringent”, “broad”, and “histone” settings, respectively) from an H3K27me3 CUT&RUN dataset using K562 cells [16] that contains broad domains by visual inspection. Remarkably, though SEACR called many fewer enriched regions than MACS2 or HOMER (28803, 97247, and 104524, respectively), SEACR regions covered more sequence (31.4 Mb) than either (28.1 Mb and 18.3 Mb), indicating that SEACR regions are broader. Indeed, the average width of SEACR regions exceeded that of MACS2 and HOMER by nearly an order of magnitude (Fig. 4a). Visual inspection of loci with broad H3K27me3 domains such as the HOXD cluster indicates that whereas MACS2 and HOMER partition the domain into several subregions, SEACR maintains the majority of the domain structure in a limited number of large signal blocks (Fig. 4b). These data indicate that SEACR is a useful tool for identifying large domains in CUT&RUN data in addition to spatially restricted binding sites.

### SEACR exhibits competitive run time and read–write memory allocation

**Fig. 5:**
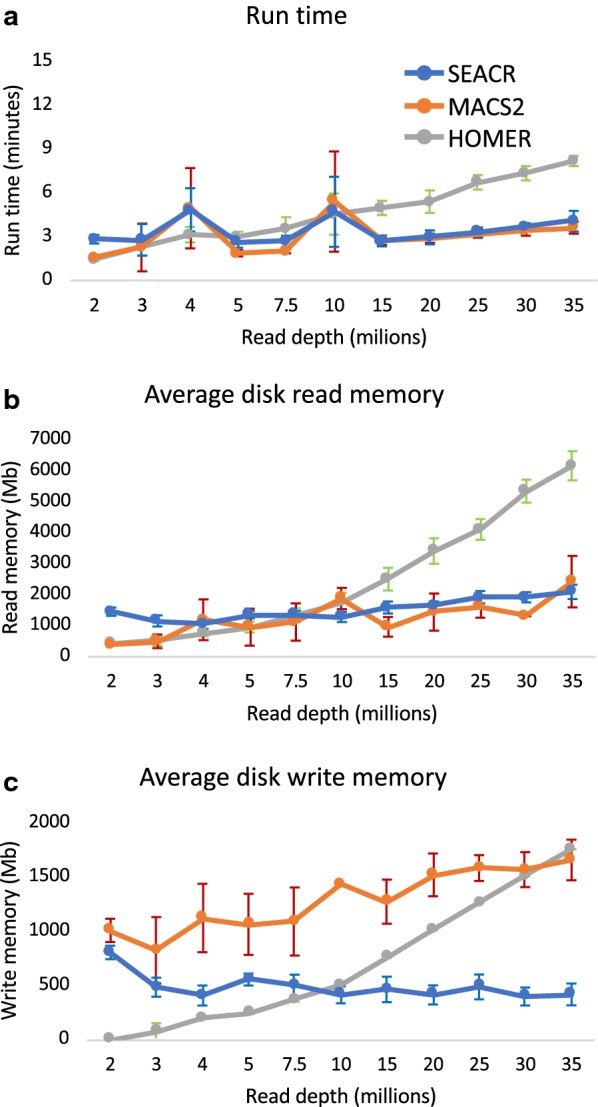
SEACR exhibits competitive run time and memory usage. Comparison of run time (**a**), average disk read memory (**b**), and average disk write memory (**c**) recorded for SEACR, MACS2, and HOMER peak calling from H3K27me3 data at read depths indicated. Error bars indicate standard deviation from the mean of 10 trials

To evaluate SEACR’s run time and memory usage in comparison with other peak callers, we measured run time, average disk read memory, and average disk write memory for instances of H3K27me3 peak calling described above. SEACR was superior or competitive with all peak callers tested in all three metrics (Fig. 5a–c). Notably, for every read subsampling level tested, SEACR finished in less than 5 min on average, demonstrating that SEACR is scalable for datasets of vastly different sizes (Fig. 5a). We conclude that SEACR is a fast and efficient method for peak calling from a range of CUT&RUN datasets.

### A web server for rapid desktop analysis using SEACR

**Fig. 6:**
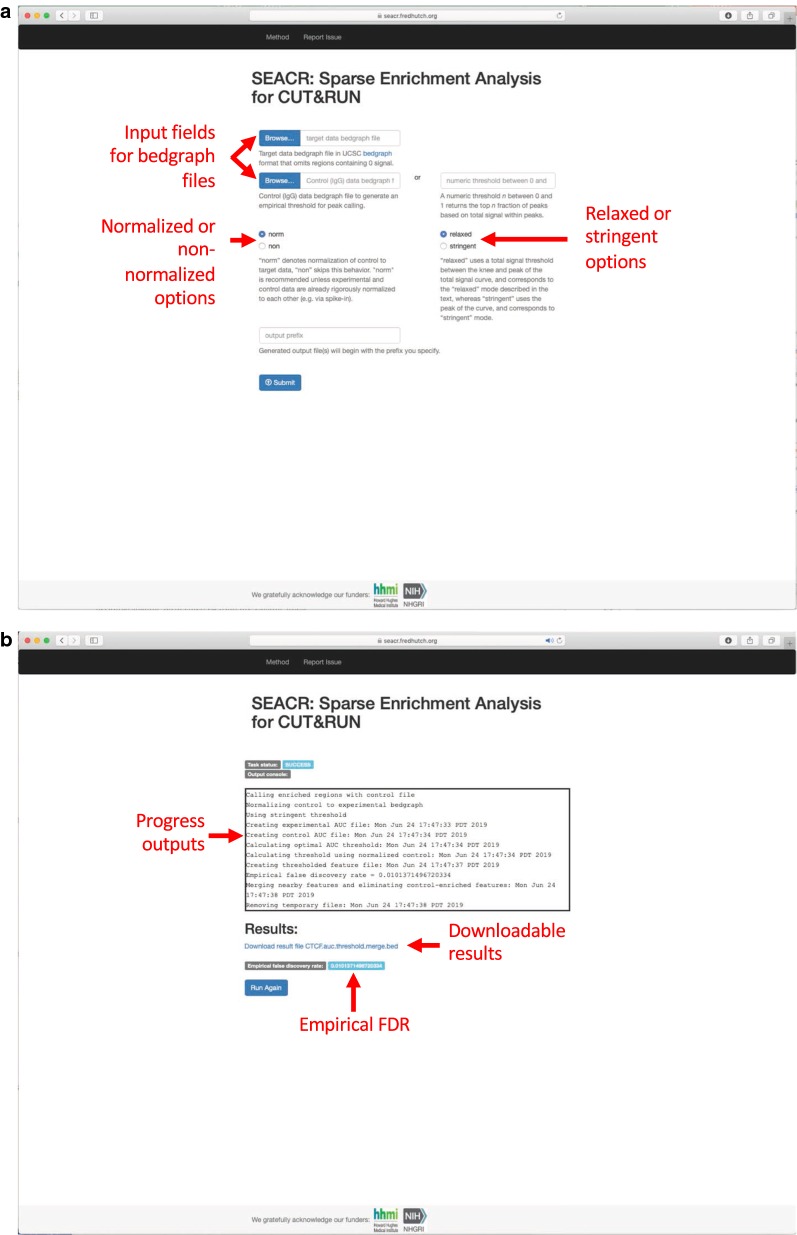
A web server for SEACR analysis. **a** The SEACR web server home page (https://seacr.fredhutch.org) contains browse functions for bedgraph input files and toggles for normalization and stringent/relaxed mode options. **b** The SEACR web server output page features a progress window with regular updates, a link for downloadable results in BED format, and the empirical false discovery rate (FDR) calculated by quantifying the percentage of control signal blocks remaining out of the total above the threshold

In the interest of disseminating SEACR as widely as possible to users with limited expertise in bioinformatics, we developed a SEACR web server (http://seacr.fredhutch.org) for plug-and-play analysis of CUT&RUN data (Fig. 6a, b). The web server accepts bedgraph files of up to 500 Mb as inputs, enables users to toggle desired parameters (normalized or non-normalized mode, and stringent or relaxed mode), reports progress during run time, and provides a link to a downloadable BED file containing the results. We anticipate that the SEACR web server will make rapid CUT&RUN analysis accessible for the broader community.

## Discussion

We have introduced a novel peak calling strategy that takes advantage of the precise position and fragment spanning information that is present in CUT&RUN data. Popular peak calling programs were designed around ChIP-seq data, where fragment spans are lacking owing to the widespread use of sonication and single-end sequencing. In contrast, our SEACR algorithm finds peaks in CUT&RUN data with a better precision–recall trade-off than the most popular ChIP-inspired peak callers. The near absence of false positives called by SEACR for Sox2 and FoxA2 transcription factors in cells that do not express them confirms the very high accuracy of CUT&RUN, in contrast to ChIP-seq, where reports of “Phantom Peaks” and other issues undermine confidence in peak calls [12, 17, 18]. SEACR is also likely to be useful for CUT&Tag [19] and other epigenomic datasets that capture fragment position and length information with high signal to noise. We expect that as the value of precise fragment information becomes better appreciated, for example in inferring chromatin dynamics [20], our block aggregation strategy will become increasingly powerful. The fast run times and favorable read memory allocation requirements have made it possible for us to offer SEACR as a public web server, which enables researchers to conveniently analyze their own data without requiring computational skills or the availability of institutional resources.

## Conclusions

SEACR is a highly specific peak caller for CUT&RUN data that outperforms common ChIP-seq peak calling algorithms in avoiding known false positives from a gold standard dataset, while exhibiting competitive or superior performance in calling peaks from diverse CUT&RUN datasets.

## Methods

### CUT&RUN

CUT&RUN was performed as previously described [10]. Briefly, cells were washed with Wash Buffer (20 mM HEPES pH 7.5, 150 mM NaCl, 0.5 mM spermidine and one Roche Complete protein inhibitor tablet per 50 mL), bound to Concanavalin A-coated magnetic beads and incubated with primary antibody diluted in wash buffer containing 0.05% digitonin (Dig Wash) overnight at 4 °C. Cells were then washed and incubated with protein A-MNase (pA-MN) for 1 h at 4 °C. Slurry was washed again and placed on an ice-cold block and incubated with Dig Wash containing 2 mM CaCl_2_ to activate pA-MN digestion. After digestion for 30 min, one volume of 2× stop buffer (340 mM NaCl, 20 mM EDTA, 4 mM EGTA, 0.05% Digitonin, 0.05 mg/mL glycogen, 5 µg/mL RNase A, 2 pg/mL heterologous spike-in DNA) was added to stop the reaction, and fragments were released by 30-min incubation at 37 °C. Samples were centrifuged 5 min at 16,000×*g*, and supernatant was recovered and DNA extracted via phenol–chloroform extraction and ethanol precipitation. Resulting DNA was used as input for library preparation as previously described [10]. Antibodies used for CUT&RUN in this study were as follows: rabbit anti-Sox2 (Abcam ab92494); rabbit anti-FoxA2 (Millipore 07-633); Guinea-Pig anti-rabbit IgG (antibodies online ABIN101961); rabbit anti-H3K4me2 (Millipore 07-030); rabbit anti-H3K4me3 (Active Motif 39159); rabbit anti-H3K27me3 (Cell Signaling Technologies CST9733); and rabbit anti-CTCF (Millipore 07-729).

### SEACR design and methodology

SEACR was designed to call enriched regions from sparse CUT&RUN data, in which background is dominated by “zeros” (i.e., regions with no read coverage). SEACR takes as input the following five fields: (1) target data bedgraph file in UCSC bedgraph format (https://genome.ucsc.edu/goldenpath/help/bedgraph.html) that omits regions containing 0 signal; (2) control (IgG) data bedgraph file; (3) “norm” denotes normalization of control to target data, “non” skips this behavior; (4) “relaxed” uses a total signal threshold between the knee and peak of the total signal curve and corresponds to the “relaxed” mode described in the text, whereas “stringent” uses the peak of the curve and corresponds to “stringent” mode; (5) prefix for output file.

Briefly, for each input bedgraph, we concatenated each region with adjacent regions to generate “signal blocks” that span all concatenated component regions and calculated total signal for each signal block by taking the sum across all component regions of the region length (region end minus region start) multiplied by its bedgraph signal (column 4):

$$t = \mathop \sum \limits_{i = 1}^{n} \left( {{\text{end}}_{i} - {\text{start}}_{i} } \right)*\left( {{\text{signal}}_{i} } \right)$$

We designated maximum signal for each signal block as the maximum bedgraph signal value for any component region contained in the block. For normalization, we generated total signal density plots for all signal blocks from target and control data, identified the total signal AUC values that corresponded to the density peak of each plot and multiplied total signal values for all control signal blocks by a “scaling factor” calculated by dividing the density peak total signal value for target data by the density peak total signal value for control data. To determine the total signal threshold *t*, we identified the value corresponding to the maximum value of *F* for the following function:

$${\text{for}}\quad t \in \left\{ {0, \ldots ,T} \right\}; F = \frac{{\mathop \sum \nolimits_{i = 1}^{m} [r_{i} > 0] - \mathop \sum \nolimits_{i = 1}^{m} [r_{i} > t]}}{{\mathop \sum \nolimits_{j = 1}^{n} [s_{j} > 0] - \mathop \sum \nolimits_{j = 1}^{n} [s_{j} > t]}}$$

where *T* is the maximum total signal value in any signal block, *r*_*i*_ is the total signal for an element *i* in the set of *m* target signal blocks, *s*_*j*_ is the total signal for an element *j* in the set of *n* target or control signal blocks, and *F* is the fraction of target signal blocks divided by total signal blocks remaining above threshold *t*. Once *t* is established, all target signal blocks exceeding *t* that do not overlap a control signal block that also exceeds *t* are retained. For “relaxed” mode, we identified the negatively inflected “knee” of the curve *k* that is nearest to and less than *F*, and defined the threshold as *F* − ((*F* − *k*)/2).

### Peak calling and precision–recall analyses

MACS2 peaks were called using macs2 callpeak -f BEDPE --keep dup all, with treatment and control files. For H3K27me3, the --broad flag was added. For local lambda-inactivated peak calling, --llocal 0 was added. HOMER peaks were called by generating tag directories for target and control datasets, then using findPeaks, -style factor for TFs, H3K4me2 or H3K4me3, and -style histone for H3K27me3.

For area under the precision–recall curve analysis (AUPR), we compared CUT&RUN peak calls from SEACR, MACS2, and HOMER to stringent sets of MACS2-called ChIP-seq peaks generated by the ENCODE consortium. The ENCODE accession numbers and thresholding used for each ENCODE peak file are as follows: H3K4me2: ENCFF099LMD, −log_10_(FDR) > 10; H3K4me3: ENCFF258PHY, −log_10_(FDR) > 10; CTCF: ENCFF002DDJ, no extra thresholding; H3K27me3: ENCFF126QYP, −log_10_(FDR) > 10. We called all peaks using loose stringency parameters in order to generate full peak files with nearly 100% recall that could be subset by ranking metrics (total signal in signal block for SEACR, −log_10_(FDR) for MACS2, and peak score for HOMER) in order to derive full precision–recall curves. For SEACR, we artificially set total signal threshold to ½ the threshold corresponding to the knee of the curve described in the “relaxed” mode. For MACS2, we added the flag –*p* 0.05. For HOMER, we added the flags –F 1.0 –P 0.25 –L 1.0 –LP 0.25 –fdr 0.25. We then used custom bash and R scripts to calculate AUPR values for each dataset. Briefly, to generate values for precision calculations, we used bedtools intersect –u [21] with the CUT&RUN peak set as the –a file and the ChIP-seq reference peak set as the –b file, and for each CUT&RUN peak reported its ranking metric and whether it overlapped a ChIP-seq peak. To calculate recall, we used bedtools intersect –u with the ChIP-seq reference peak set as the –a file and the CUT&RUN peak set as the –b file, and for each ChIP-seq peak reported the lowest ranking metric of any CUT&RUN peak that overlapped it (if any). We then calculated the percentage of CUT&RUN peaks that were overlapped by a ChIP-seq peak (precision) and the percentage of ChIP-seq peaks that were overlapped by a CUT&RUN peak (recall) for every value of the ranking metric that was recorded in our analysis, plotted the precision and recall values on a curve, and calculated the area under the curve. All subsampling was performed in 10 replicates, and error bars throughout the figures represent the standard deviation of 10 trials.

For recall and precision analysis with default peak calling cutoffs, we used ENCODE H3K4me2 ChIP-seq peaks (ENCFF099LMD) meeting a −log_10_(FDR) cutoff of 10 as the test set and used bedtools intersect with the –u flag to calculate the percentage of test set regions overlapped (fraction of true positives) or the percentage of experimental peaks outside the test set (fraction of false positives). F1 scores were calculated as follows:

$$F = \frac{{2*\left( {P*R} \right)}}{P + R}$$

where *P* is precision (fraction of called peaks that are true positives) and *R* is recall (fraction of true positives from test set identified).

## Additional files

**Additional file 1: Fig. S1.** AUPR analysis for SEACR. Plot of area under the precision–recall curve (AUPR) for peaks called from H3K4me2 (A), H3K27me3 (B), or CTCF (C) CUT&RUN data by SEACR, MACS2, MACS2 with local lambda inactivated (MACS2 llocal), and HOMER, that were compared to a stringent test set of peaks called from ENCODE ChIP-seq data for each indicated target. Read subsampling levels are indicated on the X-axis.

**Additional file 2: Fig. S2.** Peak calling metrics for SEACR default thresholds. **A-B)** Table denoting the number of bases overlapped (A) or the percentage of reads in peaks (B) called by SEACR stringent mode (top row), SEACR relaxed mode (second row), MACS2 (third row), or HOMER (fourth row). Factor and cell type from which data are derived are indicated in columns and rows, respectively.

## Data Availability

All scripts associated with SEACR are provided at https://github.com/FredHutch/SEACR. The datasets supporting the conclusions of this article are available in the GEO repository (GSE126612).
