# Supplementary material for: Peak calling by Sparse Enrichment Analysis for CUT&RUN chromatin profiling
Source: Epigenetics Chromatin. 2019 Jul 12;12:42. doi: 10.1186/s13072-019-0287-4 (PMC6624997; doi:10.1186/s13072-019-0287-4)
Supplement: Supplementary file 1 — Additional file 1: Fig. S1. AUPR analysis for SEACR. Plot of area under the precision–recall curve (AUPR) for peaks called from H3K4me2 (A), H3K27me3 (B), or CTCF (C) CUT&RUN data by SEACR, MACS2, MACS2 with local lambda inactivated (MACS2 llocal), and HOMER, that were compared to a stringent test set of peaks called from ENCODE ChIP-seq data for each indicated target. Read subsampling levels are indicated on the X-axis. [file 13072_2019_287_MOESM1_ESM.pdf]

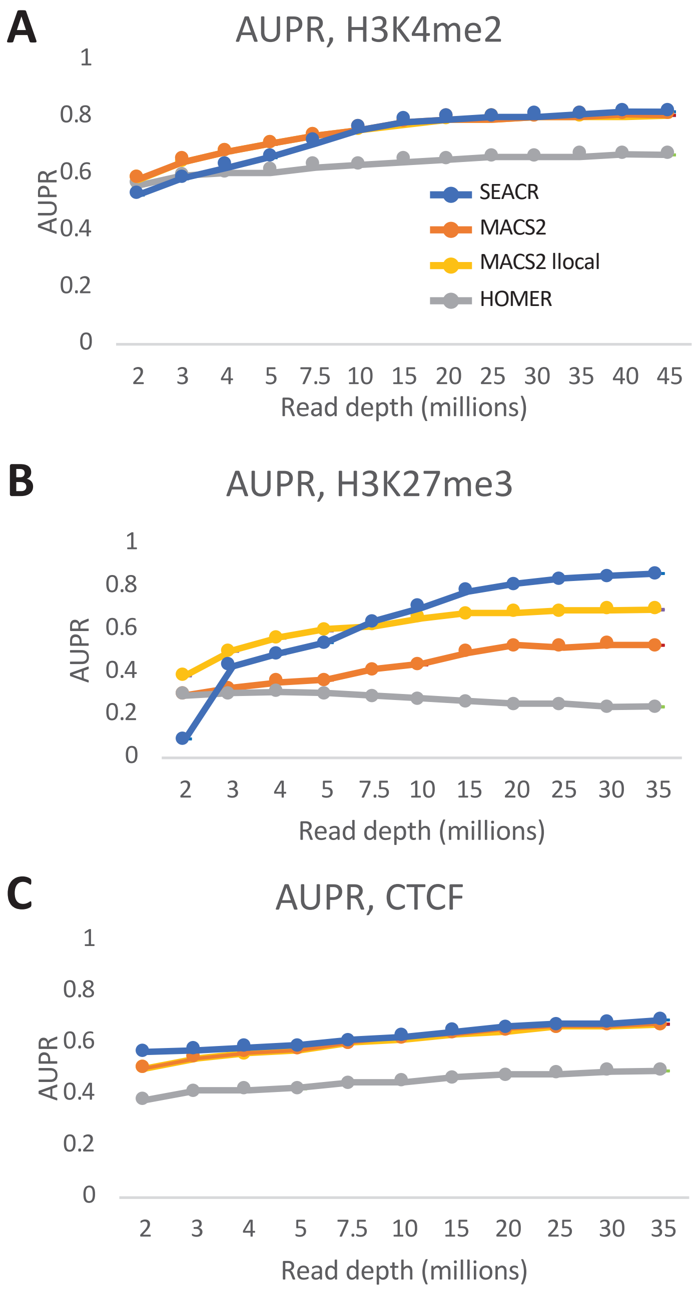

AUPR analysis for SEACR. Plot of area under the precision-recall curve (AUPR) for peaks called from H3K4me2 (A), H3K27me3 (B), or CTCF (C) CUT&RUN data by SEACR, MACS2, MACS2 with local lambda inactivated (MACS2 llocal), and HOMER, that were compared to a stringent test set of peaks called from ENCODE ChIP-seq data for each indicated target. Read subsampling levels are indicated on the X-axis.

Figure S1
