# Supplementary material for: Peak calling by Sparse Enrichment Analysis for CUT&RUN chromatin profiling
Source: Epigenetics Chromatin. 2019 Jul 12;12:42. doi: 10.1186/s13072-019-0287-4 (PMC6624997; doi:10.1186/s13072-019-0287-4)
Supplement: Supplementary file 2 — Additional file 2: Fig. S2. Peak calling metrics for SEACR default thresholds. A-B) Table denoting the number of bases overlapped (A) or the percentage of reads in peaks (B) called by SEACR stringent mode (top row), SEACR relaxed mode (second row), MACS2 (third row), or HOMER (fourth row). Factor and cell type from which data are derived are indicated in columns and rows, respectively. [file 13072_2019_287_MOESM2_ESM.pdf]

A # of Bases Covered by Peaks

SEACR (stringent)	Sox2	FoxA2
hESC	23901716	58704
Endoderm	68870	8227725

SEACR (relaxed)	Sox2	FoxA2
hESC	53639250	192624
Endoderm	214382	13342446

MACS2	Sox2	FoxA2
hESC	4608040	591963
Endoderm	463996	2770715

HOMER	Sox2	FoxA2
hESC	1058250	4050
Endoderm	7050	967950

B % of Reads in Peaks

SEACR (stringent)	Sox2	FoxA2
hESC	4.683	0.257
Endoderm	0.468	2.627

SEACR (relaxed)	Sox2	FoxA2
hESC	8.008	1.506
Endoderm	1.958	3.576

MACS2	Sox2	FoxA2
hESC	7.386	4.198
Endoderm	3.456	5.857

HOMER	Sox2	FoxA2
hESC	1.434	0.005
Endoderm	0.007	1.487

Peak calling metrics for SEACR default thresholds. A-B) Table denoting the number of bases overlapped (A) or the percentage of reads in peaks (B) called by SEACR AUC only mode (top row), SEACR Union mode (second row), MACS2 (third row), or HOMER (fourth row). Factor and cell type from which data are derived are indicated in columns and rows, respectively.

Figure S2
